# Supplementary material for: Digital Devices Use and Sleep in Adolescents: An Umbrella Review
Source: Int J Environ Res Public Health. 2025 Oct 2;22(10):1517. doi: 10.3390/ijerph22101517 (PMC12564284; doi:10.3390/ijerph22101517)
Supplement: Supplementary file 1 [file ijerph-22-01517-s001.zip › ijerph-3757125-supplementary.pdf]

Supplementary materials

Boolean search strings

MEDLINE/PubMed

("adolescents"[Title/Abstract] OR "teenagers"[Title/Abstract] OR "children"[Title/Abstract] OR "Adolescent"[Mesh] OR "Minors"[Mesh] OR "high school"[tiab]) AND

("internet"[Title/Abstract] OR "online"[tiab] OR "web"[tiab] OR "addiction"[tiab] OR "Technology Addiction"[Mesh] OR "digital"[tiab] OR "media"[tiab] OR "screen"[tiab] OR "blue light"[tiab] OR "Video Games"[Mesh] OR "videogame"[tiab] OR "gaming"[tiab] OR "games"[tiab] OR "streaming"[tiab] OR "social media"[tiab] OR "Social Media"[Mesh] OR "smart phone"[Title/Abstract] OR "smartphone"[Title/Abstract] OR "mobile phone"[Title/Abstract] OR "tablet"[tiab] OR "mobile device"[tiab] OR "console"[tiab] OR "computer"[tiab]) AND

("sleep"[Title/Abstract] OR "insomnia"[tiab] OR "Sleep Wake Disorders"[Mesh] OR "sleepiness"[tiab]) AND

(meta-analysis[Filter] OR systematicreview[Filter] OR "systematic review"[tiab])

SCOPUS

(TITLE-ABS (adolescents OR teenagers OR children OR {high school})) AND

(TITLE-ABS (internet OR online OR web OR addiction OR digital OR media OR screen OR {blue light} OR {Video Games} OR videogame OR gaming OR games OR streaming OR {social media} OR {smart phone} OR smartphone OR mobile OR tablet OR console OR computer)) AND

(TITLE-ABS (sleep OR insomnia OR sleepiness)) AND

(TITLE-ABS (meta-analysis OR {meta analysis} OR {systematic review}))

WEB OF SCIENCE

(TI=(adolescents OR teenagers OR children OR "high school") OR AB=(adolescents OR teenagers OR children OR "high school")) AND
(AB=(internet OR online OR web OR addiction OR digital OR media OR screen OR "blue light" OR "Video Games" OR videogame OR gaming OR games OR streaming OR "social media" OR "smart phone" OR smartphone OR mobile OR tablet OR console OR computer) OR TI=(internet OR online OR web OR addiction OR digital OR media OR screen OR "blue light" OR "Video Games" OR videogame OR gaming OR games OR streaming OR "social media" OR "smart phone" OR smartphone OR mobile OR tablet OR console OR computer)) AND
(TI=(sleep OR insomnia OR sleepiness) OR AB=(sleep OR insomnia OR sleepiness)) AND
(AB=(meta-analysis OR "meta analysis" OR "systematic review") OR TI=(meta-analysis OR "meta analysis" OR "systematic review"))

APA PsycArticles

Any Field: sleep *OR* **Any Field**: insomnia *OR* **Any Field**: sleepiness *OR* **MeSH**: "Sleep Wake Disorders" *AND* **Any Field**: internet *OR* **Any Field**: online *OR* **Any Field**: addiction *OR* **Any Field**: digital *OR* **Any Field**: media *OR* **Any Field**: screen *OR* **Any Field**: "blue light" *OR* **Any Field**: "Video Games" *OR* **Any Field**: videogame *OR* **Any Field**: gaming *OR* **Any Field**: games *OR* **Any Field**: streaming *OR* **Any Field**: "social media" *OR* **Any Field**: "smart phone" *OR* **Any Field**: smartphone *OR* **Any Field**: mobile *OR* **Any Field**: tablet *OR* **Any Field**: console *OR* **Any Field**: computer *AND* **Methodology**: Systematic Review *OR* Meta Analysis *AND* **Age Group**: School Age (6-12 yrs) *OR* Adolescence (13-17 yrs) *OR* Young Adulthood (18-29 yrs)

Table S1. Instruments used to assess exposure and outcomes.

**Instruments to assess the exposure**	**Instruments used to assess the outcome**
Adapted Mobile Monitoring And Intervention (Mmi)	Actigraphy
Ecological Momentary Assessment (Ema)	Child And Adolescent Sleep Checklist (Casc)
Global School-Based Student Health Survey (Gshs)	Diagnostics Interview Questions (Sqi)
Internet Addiction Scale (Ias).	Electroencephalogram (Eeg)
Internet Addiction Test (Iat)	Epworth Sleepiness Scale (Ess)
Korean Internet Addiction Scale	General Health Questionnaire- 12 (Ghq-12)
Logger App	Insomnia Questionnaire
Multiple Mini-Interview (Mmi)	Insomnia Severity Index (Isi)
Parent-Reported Questionnaire Data	Multidimensional Sub-Health Questionnaire Of Adolescents (Msqa)
Prebedtime Behaviours (Pbbs)	Munich Chronotype Questionnaire
Psacog (Presleep Arousal Scale)	Pittsburgh Sleep Quality Index (Psqi)
Self-Reported Questionnaire Data	Polysomnography (Psg)
Shortform Smartphone Addiction Inventory (Ssai).	Salzburger State Reactance Scale (Ssr Scale)
Social Network Site Adolescents Questionnaire (Snsaq)	School Sleep Habits Survey (Sshs)
Social-Media Networking Dimension Of The Socio-Digital Participation Inventory (Sdpi)	Self-Reported Questionnaire Data
Technology Use Questionnaire (Tuq)	Semi-Structured Inquiry On Sleep
Time Use Diaries (Tud)	Sleep Diary
Young'S Internet Addiction Test (Iat)	Sleep Log Based On Pittsburgh Sleep Quality Index (Psqi)
	Sleep Self-Report (Ssr)
	Structured Inquiry On Sleep
	Swiss Health Survey

Table S2. AMSTAR-2 assessment.

	Bartel et al.	Kokka et al.	Lund et al.	Da Silva et al.	Dibben et al.	Pagano et al.	Gale et al.
Did the research questions and inclusion criteria for the review include the components of PICO/PECOS?	Yes	Yes	Yes	Yes	Yes	Yes	Yes
Did the report of the review contain an explicit statement that the review methods were established prior to the conduct of the review and did the report justify any significant deviations from the protocol?*	No	No	No	Yes	Yes	Yes	Yes
Did the review authors explain their selection of the study designs for inclusion in the review?	Yes	Yes	Yes	Yes	Yes	Yes	Yes
Did the review authors use a comprehensive literature search strategy?*	Yes	Yes	Yes	Yes	Yes	Yes	Yes
Did the review authors perform study selection in duplicate?	No	Yes	Yes	Yes	Yes	Yes	Yes
Did the review authors perform data extraction in duplicate?	No	Yes	Yes	Yes	Yes	Yes	Yes
Did the review authors provide a list of excluded studies and justify the exclusions?	No	No	No	No	No	Yes	No
Did the review authors describe the included studies in adequate detail?*	Yes	Yes	Yes	Yes	Yes	Yes	Yes
Did the review authors use a satisfactory technique for assessing the risk of bias (RoB) in individual studies that were included in the review?*	No	Yes	Yes	Yes	Yes	Yes	Yes
Did the review authors report on the sources of funding for the studies included in the review?	No	No	No	No	No	No	No
If meta-analysis was performed, did the review authors use appropriate methods for statistical combination of results?*	Yes	N.A.	N.A.	N.A.	N.A.	Yes	N.A.
If meta-analysis was performed, did the review authors assess the potential impact of RoB in individual studies on the results of the meta-analysis or other evidence synthesis?*	No	N.A.	N.A.	N.A.	N.A.	Yes	N.A.
N.Did the review authors account for RoB in primary studies when interpreting/discussing the results of the review?*	No	Yes	Yes	Yes	Yes	Yes	Yes
Did the review authors provide a satisfactory explanation for, and discussion of, any heterogeneity observed in the results of the review?	No	No	No	Yes	Yes	Yes	Yes
If they performed quantitative synthesis, did the review authors carry out an adequate investigation of publication bias (small study bias) and discuss its likely impact on the results of the review?*	No	N.A.	N.A.	N.A.	N.A.	Yes	N.A.
Did the review authors report any potential sources of conflict of interest, including any funding they received for conducting the review?	Yes	Yes	Yes	Yes	Yes	Yes	Yes
Total of yes	6	9	9	11	11	15	11
*Critical flaws	5	1	1	0	0	0	0

Rating overall confidence	Very low	Low	Low	Moderate	Moderate	Moderate	Moderate

Table S3. Strength of evidence assessment following the GRADE approach.

Factor	Total	Limitations in study design or execution (risk of bias)	Indirectness of evidence	Inconsistency of results	Imprecision	Publication bias	Large magnitude of effect	Effect of plausible residual confounding	Dose-response gradient
**Total Sleep duration**		↓ 1 or 2 levels	↓ 1 or 2 levels	↓ 1 or 2 levels	↓ 1 or 2 levels	↓ 1 or 2 levels	↑ 1 or 2 levels	↑ 1 level	↑ 1 level
Bartel et al.	Low	-2	0	0	-1	0	0	0	1
Kokka et al.	Moderate	-1	0	-1	0	0	0	0	1
Lund et al.	Moderate	-1	0	-1	0	0	0	0	1
Da Silva et al.	Moderate	0	-1	-1	0	0	0	0	1
Dibben et al.	Moderate	-1	0	0	-1	0	0	0	1
Pagano et al.	Moderate	0	-1	-1	0	0	0	0	1
Gale et al., 2025	Moderate	0	-1	0	-1	0	0	0	1
Bedtime procrastination									
Bartel et al.	Low	-2	0	0	-1	0	0	0	1
Kokka et al.	Moderate	-1	0	-1	0	0	0	0	1
Lund et al.	Moderate	-1	0	-1	0	0	0	0	1
Da Silva et al.	Moderate	0	-1	-1	0	0	0	0	1
Dibben et al.	Moderate	-1	0	0	-1	0	0	0	1
Pagano et al.	Moderate	0	-1	-1	0	0	0	0	1
Gale et al., 2025	Moderate	-1	0	0	-1	0	0	0	1
Sleep quality									
Da Silva et al.	Moderate	0	-1	-1	0	0	0	0	1
Kokka et al.	Moderate	-1	0	-1	0	0	0	0	1
Lund et al.	Moderate	-1	0	-1	0	0	0	0	1
Dibben et al.	Moderate	-1	0	0	-1	0	0	0	1

Pagano et al.	Moderate	0	-1	-1	0	0	0	0	1
Gale et al., 2025	Moderate	-1	-1	0	0	0	0	0	1
